# Novel High Content Screen Detects Compounds That Promote Neurite Regeneration from Cochlear Spiral Ganglion Neurons

**DOI:** 10.1038/srep15960

**Published:** 2015-11-02

**Authors:** Donna S. Whitlon, Mary Grover, Sara F. Dunne, Sonja Richter, Chi-Hao Luan, Claus-Peter Richter

**Affiliations:** 1 Department of Otolaryngology Head and Neck Surgery, Northwestern University, Chicago, IL, 60611; 2 Knowles Hearing Center, Northwestern University, Evanston IL, 60208-3550; 3 Interdepartmental Neurosciences Program, Northwestern University, Chicago, IL, 60611-3010; 4 High Throughput Analysis Laboratory, Northwestern University, Evanston, IL 60208; 5 Department of Biomedical Engineering, Northwestern University, Evanston, IL 60208.

## Abstract

The bipolar spiral ganglion neurons (SGN) carry sound information from cochlear hair cells to the brain. After noise, antibiotic or toxic insult to the cochlea, damage to SGN and/or hair cells causes hearing impairment. Damage ranges from fiber and synapse degeneration to dysfunction and loss of cells. New interventions to regenerate peripheral nerve fibers could help reestablish transfer of auditory information from surviving or regenerated hair cells or improve results from cochlear implants, but the biochemical mechanisms to target are largely unknown. Presently, no drugs exist that are FDA approved to stimulate the regeneration of SGN nerve fibers. We designed an original phenotypic assay to screen 440 compounds of the NIH Clinical Collection directly on dissociated mouse spiral ganglia. The assay detected one compound, cerivastatin, that increased the length of regenerating neurites. The effect, mimicked by other statins at different optimal concentrations, was blocked by geranylgeraniol. These results demonstrate the utility of screening small compound libraries on mixed cultures of dissociated primary ganglia. The success of this screen narrows down a moderately sized library to a single compound which can be elevated to in-depth *in vivo* studies, and highlights a potential new molecular pathway for targeting of hearing loss drugs.

The bipolar cochlear spiral ganglion neurons (SGN) carry auditory information from the cochlea to the brain. These neurons, whose organized connections in the cochlea and the brain represent sound frequencies, are essential for preserving normal hearing, but are vulnerable to acoustic insult, toxicity and aging^1^,^2^,^3^,^4^,^5^,^6^. When SGN are damaged or die, hearing impairment or deafness results. Synapses between peripheral nerve fibers of the SGN and the hair cell receptors in the cochlea are the first to degenerate, followed by the retraction of the fiber over various time periods and finally neuronal cell death^2^,^3^. After neurite retraction, spontaneous regrowth of peripheral fibers back to the hair cell region of the cochlea is limited. However, the connections to the brain stem retain a generally organized frequency representation even when the peripheral fibers have retracted. Regeneration of the peripheral neurites is a goal in hearing science, but the biochemical pathways that prevent spontaneous neurite regeneration from SGN are largely unknown. Consequently, no FDA approved drugs exist to promote neurite regeneration from SGN^7^.

*In vitro* and/or *in vivo* studies indicate that certain biologic compounds such as BDNF^8^,^9^,^10^,^11^,^12^, BMP2^13^, NT3^14^, GDNF^15^, FGF8a^16^ and erythropoietin^17^ promote neurite initiation, total neurite branch length, maintenance of myelin rings, length of the neurite from the edge of an explant, and/or number of neurites in the epithelium of damaged cochleas. However, these biologics initiate complex, downstream molecular signaling pathways that are ill-defined in the cochlea. In addition, the proteins are difficult to consistently maintain in the ear. Assorted studies have also attempted to look more closely at molecular mechanism^18^,^19^,^20^,^21^,^22^,^23^,^24^,^25^,^26^,^27^,^28^,^29^, but the variety of models (dissociated cells, spiral ganglion explants, deafened animals) as well as the range of experimental measurements and the variety of morphologies analyzed makes them difficult to compare and to relate to the problem of regenerating the uninterrupted length of an SGN neurite.

Initial screening of new compounds in deaf animal models is impractical due to the time and resources required for deafening, physiologic measurements, anatomical assays and hearing testing. In view of these difficulties as well as the dearth of information on SGN neurite length regeneration mechanisms and the lack of FDA approved drugs for regenerating cochlear neurites, a fresh approach to the problem was necessary. With the goal of jumpstarting the process of drug discovery for the cochlea, we designed an original phenotypic assay using primary, mixed cultures of dissociated mouse spiral ganglia. We applied this assay to screen 440 components of the NIH Clinical Collection (NCC), a library of compounds with a history of use in clinical trials. The screen detected regenerative activity of the HMG-CoA reductase inhibitor, cerivastatin, on SGN neurite length and showed that it could be blocked by the simultaneous application of geranylgeraniol. These results demonstrate the utility of screening small compound libraries on mixed cultures of dissociated primary ganglia. The success of this screen narrows down the potential compounds and their reactive pathways to one which can more effectively be examined in depth in a deaf animal model. The work implicates the geranylgeranyl-pyrophosphate branch of the mevalonate pathway in the regulation of SGN neurite growth and draws attention to a potential new molecular pathway for targeting of hearing loss drugs.

## Results

### Validation of the SGN neurite length assay

**Figure 1:**
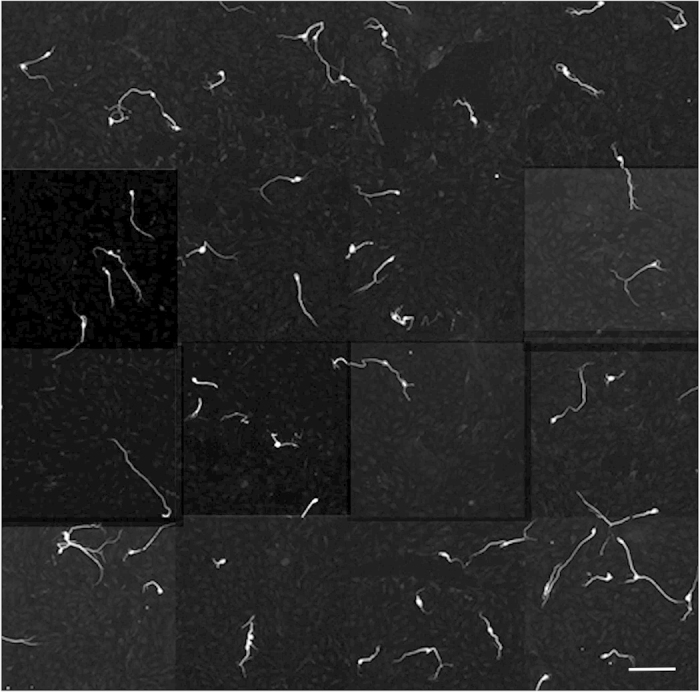
Representative example of a culture of dissociated spiral ganglia in one well of a 384 well plate. After 42 hours, culture was fixed, then immunolabeled for the neuronal marker βIII tubulin. The well was then automatically imaged at 10x, 16 fields per well (about 80% of the surface area) on an ArrayScan VTi imager, then montaged to show the pattern of neuron morphologies. In control cultures, neurons are mainly monopolar, with some bipolar neurons. Very few neurite free or multipolar neurons are detected. Neurite lengths are non-uniform. Schwann cells, fibrocytes and other non-neural cells covering the entire field are unlabeled and therefore invisible in this image. Scale bar: 200 μm.

**Figure 2:**
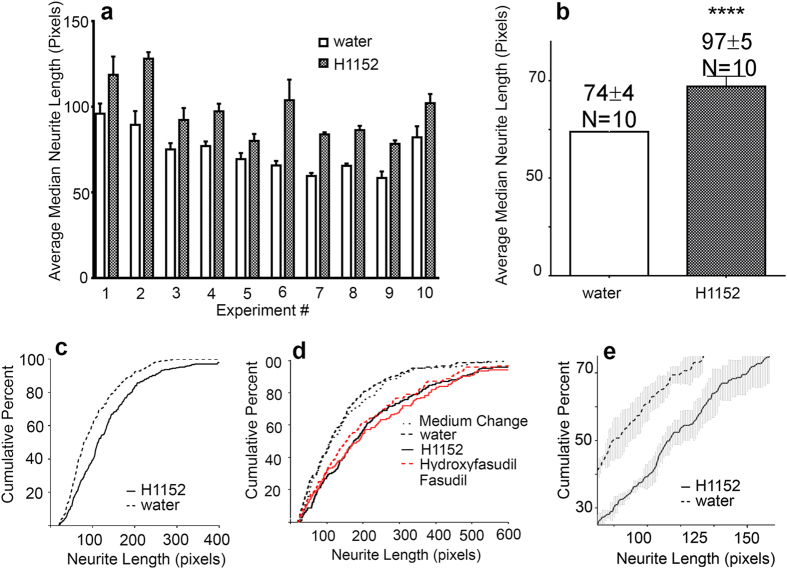
Validation of a novel assay for spiral ganglion neurite length. (**a**) Individual experimental measurements of the average median neurite lengths (raw data, in pixels) from each of ten experiments, each plated in quadruplicate in 384 well plates. In every experiment, H1152 elicits longer neurites. (**b**) Average of the median neurite lengths from all 10 experiments in A. Bars represent SEM. ****p < .0001. (**c**) Example of cumulative percent histograms of neurite lengths from both the positive (H1152) and negative (water) control wells. These are the controls from plating depicted in Fig. 4e. Histograms from quadruplicate wells were averaged. For clarity, SEM is omitted. (**d**) Cumulative percent histograms of neurite lengths from control cultures and those treated with two other Rho kinase inhibitors, fasudil and hydroxyfasudil. Histograms from quadruplicate wells were averaged. Cultures for this experiment were prepared from postnatal day 2 animals and were maintained in BMP4 medium for 4 days. For clarity, SEM is omitted. Inhibition of Rho kinase by H1152, Fasudil (100 μM) or Hydroxyfasudil (100 μM) increases population neurite length as indicated by the rightward shift of all three overlapping graphs. Histograms from cultures treated with water or with a simple medium change show shorter neurite lengths, as indicated by the overlapping graphs higher and to the left. (**e**) Example of the expansion of region containing the 25–75^th^ percentile of the positive control in graph C, where the largest differences between positive and negative controls are detected.

Chemical library screens are usually carried out on cell lines or easily accessible and plentiful neurons with the hope that the findings will transfer to animal and human studies in specific tissues. To lessen the possibility that our findings would not reproduce in intact spiral ganglia, a priority in this work was to carry out the *in vitro* screen on a cellular substrate that was as close as practically possible to the cells we aim to target *in vivo*. For this, we chose the spiral ganglia of newborn mice, which are accessible for dissection and whose neurons survive in culture. We also opted to use mixed cultures of the ganglia because neurites do not naturally grow in isolation and the ganglia provides cells that are usually present in the vicinity of neurons and neurites – Schwann cells and fibroblasts – which are known to exert effects on neuronal survival and growth. At birth, the majority of SGN peripheral fibers have already reached their hair cell receptors^30^. When newborn spiral ganglia are dissociated, the neurons lose their nerve fibers^31^, but in culture, neurites regrow. In a typical culture, neurons coexist together with Schwann cells and fibroblasts^31^,^32^, but in Fig. 1, a montage of 16 images of a typical culture, immunolabeling for neuron specific βIII tubulin highlights only the neurons. Since the ultimate goal for regenerative therapy is to promote extension of peripheral fibers of sufficient length to re-contact the target organ, we focused our assay solely on measuring the longest continuous distance from the cell soma to the end of the neurite. Nonetheless, the images are stored and can, in the future, be used for other measurements. In our past experiments, we manually imaged the cultures and measured neurites from the images^21^,^33^. To measure tens of thousands of neurites that we will acquire in a screen, we developed an automated imaging protocol in combination with software assisted measurements of the longest neurite per neuron. The automated measurements were validated by manual measurements^21^. We found that in SG cultures prepared from newborn mouse cochleas, the population of neurites exhibits a range of lengths, which is increased by exposure to the Rho kinase inhibitor H1152^21^. We used H1152 treatment to validate our automated assay. The averaged median neurite lengths in quadruplicate H1152 cultures in 10 separate experiments (Fig. 2a,b), were significantly longer than the lengths in the corresponding vehicle treated control cultures(****p < .0001). The range of and consistency of neurite lengths in cultures can be better appreciated by comparing cumulative percent histograms of neurite lengths (Fig. 2c, maintained in standard medium containing BDNF and NT3). Cultures with the longest populations of neurites plot toward the right of the graph, shorter, to the left We also evaluated the effect of H1152 and two other Rho kinase inhibitors in cultures prepared from postnatal day 2 animals and maintained for 4 days with medium that also contained BMP4, a morphogen that normally results in shorter neurites^33^. Inhibition of Rho kinase by H1142, fasudil or hydroxyfasudil stimulated regeneration of neurite length. Thus the assay was consistent and reproducible even with cultures prepared from older animals and maintained in a separate medium for a longer (4 days vs 42 hours) assay period. For the screen below, the cumulative percent histograms averaged from the four replicate wells of the positive control (H1152) were further enlarged in the area of the 25^th^ to 75^th^ percentile to emphasize the region of the largest differences between positive and negative controls and to minimize the contributions of exceptionally long or short neurite outliers (Fig. 2e is derived from Fig. 2c). All averaged histograms from control and candidate compound tested wells were then plotted on this same axis for each plate.

### Screening the NCC for promotion of neurite length regeneration

**Figure 3:**
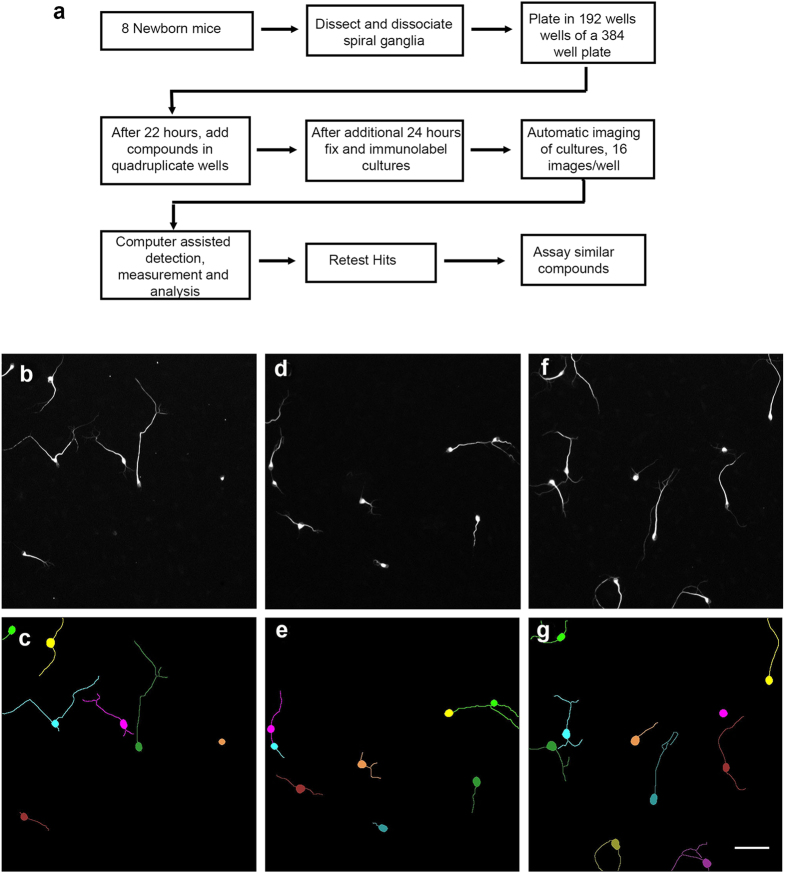
Flow chart for screen and examples of neuron/neurite detection by software. (**a**) Sixteen cochleas are sufficient to plate 192 wells of a 384 well plate. Each condition is tested in quadruplicate and each plate has its own positive and negative controls. Automatic imaging on the ArrayScan VTi provides 16 non-overlapping images per well (about 80% of the surface area). Neurites are detected and measured by the software HCA Vision. Only neurites longer than 20 pixel length, about the diameter of the neuron, are measured. (**b**–**g**) Example photographic fields (**b**,**d**,**f**) and corresponding depiction of software detected neurons and all segments of the neurites (**c**,**e**,**g**). The neurites are color coded to their neurons. Very occasionally, neurites are incorrectly segmented as in the upper right pair in E, although it does not seem to interfere with reproducible detection of neurite elongating activity. The longest, continuous path of a neurite from the cell body to the end of the neurite is calculated for each neuron. Scale: 100 μm.

Compounds in the NCC library each have a history of use in clinical trials and thus a background of information on structures, human toxicity and often biological targets. For the screen, 440 compounds of the NCC were evaluated as depicted in the flow chart in Fig. 3a^21^,^31^,^33^ with automated imaging (Fig. 3b,d,e) and software assisted (HCA Vision) neurite detection (Fig. 3c,d,f). Based on the above and prior studies^21^, H1152 (16.6 μM) was incorporated as a positive control, DMSO (<0.2%; the carrier for the candidate compounds) and water (<0.2%, the carrier for H1152) as the negative controls. All candidate compounds were assayed at 8.3 μM in medium containing neurotrophins and serum. In addition, because we showed previously that bone morphogenetic protein 4 (BMP4) inclusion decreases neurite lengths^33^, a subset of the library, 160 compounds, was also re-assayed in medium containing BMP4. All compounds, as assayed, are listed in the Supplemental Materials.

**Figure 4:**
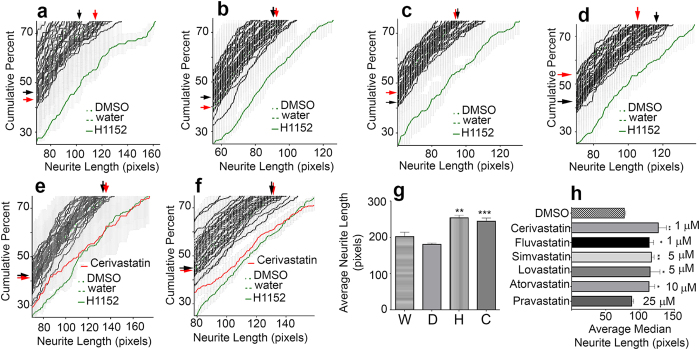
Statins stimulate neurite elongation from spiral ganglion neurons. (**a**–**f**) Examples of cumulative percent histograms of cultures from 6 separate platings. SEM from quadruplicate wells (gray) are plotted for each treatment. Histograms of cultures with tested compounds (black) are plotted atop those from cultures treated with DMSO or water (negative controls) and H1152 (positive control), green. The negative control graphs cannot be seen because the graphs from cultures treated with chemical compounds overlie them. (**a**–**e**) Represent a total of 200 tested compounds (5 dissections) assayed in medium without BMP4. (**f**) represents 40 compounds assayed in medium with BMP4. One compound, cerivastatin (red) causes a shift to the right of the histograms in both media, indicating populations of longer neurites. (**a–f**) black arrows, edges of the hidden DMSO control graph; red arrows, edges of the hidden water control graph. (**g**) Average neurite lengths of the 10 longest neurites from quadruplicate wells of 5 experiments. D, DMSO; W, water; H, H1152; C, Cerivastatin. Average lengths in H1152 and Cerivastatin cultures are longer than those in water or DMSO. Statistical analysis by ANOVA. **p < .01, H1152 compared to water. **p < .001 Cerivastatin compared to DMSO. (**h**) Average median neurite lengths from quadruplicate cultures treated with the indicated statins, depicted at their lowest effective concentrations. All tested statins, except pravastatin, increase neurite lengths at concentrations below 10 μM. *p < .05; **p < .01 There is no statistically significant change in neurite length in cultures treated with pravastatin up to 25 μM.

As representative of the screen, Fig. 4a–f illustrate results from six separate platings. Five graphs (4a–4e) depict results (a total of 200 compounds) in cultures without BMP4. One graph (Fig. 4f) depicts results (40 compounds) for cultures additionally containing BMP4. The histograms for compounds that do not stimulate neurite growth overlie those of the vehicle alone (DMSO or water) treated cultures, forming a band of histograms in the upper left of each graph. A “hit” was defined as a compound that increased population neurite lengths as least as much as the positive control. The results were consistent regardless of the medium. From the NCC, only the HMG-CoA reductase inhibitor cerivastatin (Fig. 4e,f) increased neurite length. Cerivastatin (10 μM) applied together with H1152 (16.6 μl) did not increase neurite length over that in cultures exposed to H1152 alone (data not shown). To ensure that the rightward shift of the histograms in the presence of H1152 and cerivastatin reflected actual increases in neurite length rather than a loss of neurons with short neurites, the longest 10 neurites were averaged in each well, across replicates, and across 5 experiments. The average length increased significantly in the presence of cerivastatin or H1152 over that in DMSO or water (vehicle treated) cultures (Fig. 4g).

Other statins increased neurite length. Cerivastatin, fluvastatin, lovastatin, simvastatin and atorvastatin each increased neurite lengths at concentrations at or below 10 μM, although the lowest effective concentrations differed (Fig. 4h). Cerivastatin and fluvastatin were most potent. Only pravastatin failed to increase neurite length - even at concentrations up to 25 μM.

**Figure 5:**
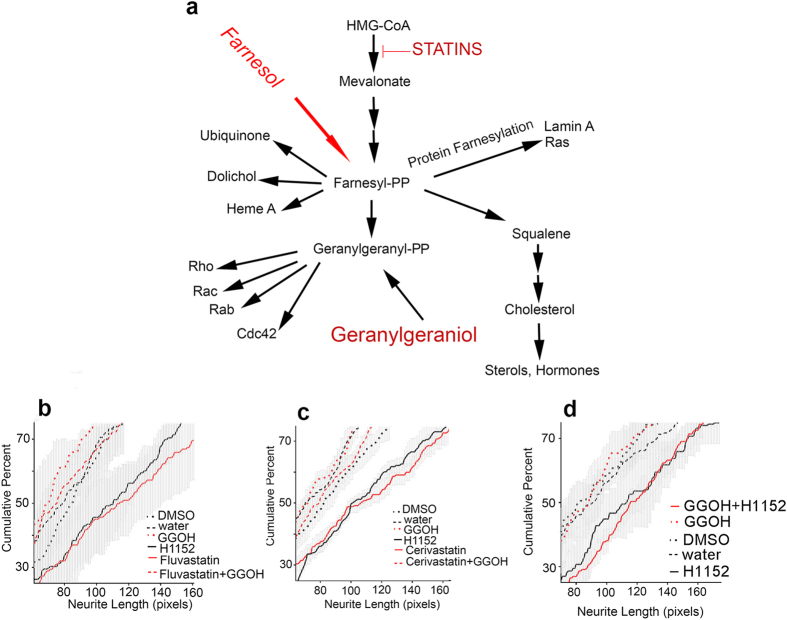
Schematic of the Mevalonate Pathway and Effects of Geranylgeraniol. HMG-CoA is the starting material for a variety of biological compounds. The first step in the pathway, HMG-CoA reductase is inhibited by statins. Downstream of mevalonate is a 15 carbon isoprenoid pyrophosphate, farnesyl-pyrophosphate (farnesyl-PP). Farnesyl-PP is used to synthesize compounds such as ubiquinone, dolichol, heme A, squalene and geranylgeranyl-pp. It is also used in a post translational modification, farnesylation, of a variety of proteins including Lamin A and Ras. Geranylgeranyl-PP in mammalian systems is known to be the source of a 20 carbon isoprenoid modification (geranylgeranyl group) of a number of proteins including Rho, Rac, Rab and Cdc42. Exogenous all trans geranylgeraniol can be used as a source for geranylgeranylated proteins. Exogenous farnesol can be used as a source for at least one pool of farnesyl PP, but it does not get incorporated into geranylgeranylated proteins^36^. (**b,c,d**) Cumulative percent histograms of neurite lengths from treatments in three different platings. Fluvastatin, 5 μM (**b**), Cerivastatin, 10 μM (**c**), and H1152 (**b**–**d**) increase neurite length. In b and c, the histograms of the two statins overlie that of the positive control, H1152. When added with Fluvastatin (**b**) or Cerivastatin (**c**), Geranylgeraniol (GGOH, 10 μM) blocks the statin induced increase in neurite length. Geranylgeraniol does not block the H1152 increase in neurite length (**d**).

By inhibiting HMG-CoA reductase activity, statins can influence not only cholesterol biosynthesis, but also various other downstream branches in the mevalonate pathway^34^,^35^, including one that normally synthesizes geranylgeranyl-pyrophosphate (Fig. 5a). To examine the role of this branch, the compound geranylgeraniol (all trans), which bypasses depletion of geranylgeranyl-pyrophosphate^36^, was added to the cultures at the same time as fluvastatin (Fig. 5b), cerivastatin (Fig. 5c), or H1152 (Fig. 5d). Geranylgeraniol blocked the effect of the statins on neurite extension (Fig. 5b,c) without affecting neurite extension in H1152 treated cultures (Fig. 5d). In other cells, statins also inhibit a variety of metabolic processes dependent on farnesylation by farnesyl-pyrophosphate^34^,^35^,^37^. Exogenous farnesol can serve as a precursor for sterol biosynthesis and protein farnesylation in mammalian cells, but is not converted to geranylgeranyl-pyrophosphate^36^. Farnesol (10 μM) did not block the statin induced increase in neurite elongation (data not shown) confirming that inhibition of cholesterol synthesis is not the mechanism underlying the effect of statins on neurite extension. Taken together, the results indicate that the geranylgeranyl-pyrophosphate branch of the mevalonate pathway is involved in slowing SGN neurite elongation and that statins enhance neurite extension by depleting geranylgeranyl-pyrophosphate pools.

### Neuronal survival in the presence of fluvastatin

Since these experiments were intended to measure effects on neurite extension, neuron numbers were low by design, to keep overlapping neurites to a minimum. This resulted in some variability between different experiments in the average number of neurons per culture. Nevertheless, there was no significant difference in average neuron survival (N = 9 experiments, quadruplicate wells) after addition of statins (52.36 ± 14 SEM neurons per well) compared to addition of vehicle (57.9 ± 11 SEM).

## Discussion

After cochlear insult, loss of hair cell/neuron synapses and retraction of peripheral nerve fibers, some SGN survive for long periods with their central connections largely intact. To reinstate the transfer of meaningful auditory information from the cochlea to the brain, and to repair hearing, interventions that maintain SGN and promote regrowth and reconnection of the peripheral fibers will be required. Such biological interventions would also provide an improved substrate for cochlear implant function. However, the cellular machinery that regulates neurite regeneration from damaged SGN is largely unknown. Drug discovery has been limited by this lack of understanding of SGN neurite growth, by the time and resource constraints of screening compound libraries with physiological or anatomical assays in deaf animal models and by the relative paucity of spiral ganglion neurons (in a mouse, approximately 10,000; in a human, approximately 30,000 per cochlea^38^). Consequently, there are no drugs that are FDA approved to promote regenerative responses by SGN.

In order to jumpstart a new drug discovery approach for hearing medicine, we developed a novel screening method using primary mixed cultures of mouse spiral ganglia as a substrate, automated imaging and measurements of the longest neurite per neuron, evaluation by comparisons of cumulative percent histograms of neurite lengths with positive and negative controls, and duplicate testing in cultures maintained in different media. This initial screen, using the NCC as a compound library, allowed us to focus on compounds with known toxicity profiles having known effects on human biology. We used this approach in a blinded study of 440 compounds and revealed neurite length promoting activity - in both the standard medium and BMP4 containing medium normally known to cause shorter neurites^33^ - by one compound previously unknown to stimulate spiral ganglion neurite growth, and successfully distinguished it from the 339 other compounds with no activity.

Our study was intended to address the biological problem of regeneration of SGN peripheral fibers of sufficient length to re-contact hair cells. We therefore evaluated the ability of a compound to increase the farthest reach of a neurite rather than its ability to increase the numbers of neurites or neurite branches^21^,^33^. When we discovered that our neurite promoting compound was a statin, we compared our study with published reports of statins in other areas of the nervous system. We found that in the statin literature, unambiguous measurement of the length of the longest neurite is rare, if not completely absent. Instead, the features reported as “neurite growth” vary widely and therefore the physiologies being assessed differ. For example, some studies compare the number of neurites per neuron^39^,^40^,^41^,^42^,^43^ (a measure of neurite initiation); the number of neurons with neurites^44^,^45^,^46^ (which may represent both neurite initiation and/or loss of a specific population of neurons or neurites); branch number^39^,^44^ (indicates the number of forks in the neurite, perhaps showing an increase in neurite “mass” but not, necessarily reflecting the farthest extent a neurite can grow); total neurite branch length^39^,^47^ (which adds all segments of primary neurite and each of its branches, not necessarily representative of the length of a primary neurite); total primary neurite length per neuron (which measures the sum of the distance of the farthest extent of all neurites from the cell body and is dependent on neurite number as well as length); or density of a neurite expression marker^47^ (which cannot distinguish between changes in marker expression, neurite branching, neurite length or increased neurite initiation). Our compounds are added 22 hours after plating, after the neurites have already initiated, and the sole focus is to directly quantify the longest extent a neurite can grow during the ensuing 24 hours of culture time. We are however, able in the future to return to our images whenever other types of neurite measurements are relevant to future biological questions. The results reported here, however, do add to the knowledge of statin effects on neurites by demonstrating that they increase the length from the cell body to the end of the neurite by increasing the overall rate of growth.

Our “hit”, cerivastatin, inhibits the HMG-CoA reductase, the rate limiting enzyme in the mevalonate pathway of cholesterol biosynthesis. In human medicine, HMG-CoA reductase inhibition by statins is used to lower blood cholesterol levels. However, the mevalonate pathway comprises multiple branches downstream from HMG-CoA reductase^37^ (Fig. 5a) and therefore statin inhibition can also potentially alter the regulation of a variety of non-cholesterol related enzymatic activities and gene expressions. Here, geranylgeraniol but not farnesol blocked the statin increase in SGN neurite length, indicating that cholesterol reduction does not drive neurite elongation. Rather, the statins act by depleting the pool of geranylgeranyl-pyrophosphate, downstream of HMG-CoA reductase, which was replenished by exogenous addition of geranylgeraniol but not farnesol^36^. Not all statins have identical chemical or biological properties^48^. In our study, the lowest concentrations that effectively increased neurite regeneration differed among the statins. Cerivastatin and fluvastatin were the most potent, and pravastatin, the most hydrophilic of the statins evaluated, was inactive in our assay.

These findings underscore the significance of the geranylgeranyl-pyrophosphate branch of the mevalonate pathway in slowing SGN neurite growth and highlight a potential new pathway to target by more conventional screening for hearing loss drugs. Further, they identify statins, a class of drugs already vetted in human medicine as a potential intervention to promote nerve fiber regeneration by damaged SGN. In addition, in order to screen a 440 member compound library, we have had to develop a new approach to assaying compounds for effects on SGN neurites. Although lower throughput than standard, target-based pharmaceutical screens, this one phenotypic screen assayed considerably more compounds than have been reportedly assayed in 25 years of *in vivo* and *in vitro* studies of SGN neurite growth.

Among other neuronal populations, statin effects are variable and unpredictable - protecting against^49^,^50^ or causing^41^,^42^,^51^,^52^ neuronal cell death, increasing^45^ or decreasing^44^ the number of neurite bearing cells, increasing, maintaining or degenerating neurites^39^,^40^,^42^,^44^,^53^,^54^,^55^, and increasing branch number^54^,^55^. In the single previously published study of statins and cochlear neurons, simvastatin was reported to induce cell death in cochlear neuroblasts (VOT-33 cells)^51^. Thus there was no way to predict that statins would stimulate neurite growth in our screen. Here, the strategy to develop our assay directly on primary SGN rather than on cell lines or other primary neurons worked to our advantage. We found that statins did not significantly alter SGN survival.

Depletion of geranylgeranyl-pyrophosphate pools by statins interferes with the post-translational addition of geranylgeranyl groups to proteins, a modification that is thought to help target proteins to membrane locations. When geranylgeranyl groups are removed, the proteins reside in the cytoplasm with lower, but not necessarily absent, enzymatic activities^35^. The most notable geranylgeranylated proteins are members of the Ras superfamily of small GTP binding proteins, including Rab, and certain of the subfamilies of the Rho proteins (Rho, Rac, Cdc42), although other proteins are known and predicted to undergo this posttranslational modification^56^. The Rho family proteins are involved in regulation of the actin cytoskeleton, which is critical for the process of neurite extension. We showed here and previously^21^ that inhibitors of one of the various downstream effectors of Rho, Rho kinase, also increase SGN neurite length. Therefore, lowering the activity of Rho by depleting the pool of geranylgeranyl-pyrophosphate, is one likely mechanism for mediating at least some of the effects of statins in our cultures. In support of this possibility is the observation that cerivastatin applied together with the Rho kinase inhibitor H1152 does not increase neurite lengths as compared to treatment with H1152 alone. However, regulation of the mevalonate pathway is complex, and its control can be altered both by direct effects of intermediate and end compounds on HMG-CoA reductase and by effects of these intermediates and of statins on gene expression^34^. Further, participation of geranylgeranylated proteins outside the Rho protein class is also a possibility.

The present work demonstrates a statin induced increase in neurite growth rate in mixed cultures of SGN. As a screening procedure to narrow down a large number of potential compounds to a few that can be elevated to in depth, *in vivo* evaluation, the screen does not distinguish between peripheral or central SGN fibers (which can be determined by studies *in vivo*) or identify the primary cell type(s) that the statins target, which will become important when statin protective or regenerative effects are confirmed in animal models.

Safe delivery of drugs directly to the inner ear is under study by a variety of research groups^57^. Optimally, the ability to apply time release drugs in very small doses directly to the cochlea will allow maximal drug concentration delivered directly where needed but will generate miniscule whole body concentrations, limiting systemic side effects. At present, there are so few potential compounds available for protection and repair of the inner ear that without new discoveries it will be difficult to take advantage of these developing technologies. Our unusual phenotypic screening assay has now provided a way to enlarge the knowledge of cochlear neurite regeneration promoting mechanisms and to identify new, potentially effective compounds for preventing and treating hearing loss.

## Methods

### Animals

Timed, pregnant CD-1 mice, (Charles River Laboratories, Wilmington, MA, USA), embryonic day (E) 18, on day of arrival, were allowed to deliver in the animal care facility at Northwestern University. Newborn pups of both sexes were used. Animals were cryo-anesthetized as reported^31^ before aseptic dissection. The care and use of animals in this study were carried out in accordance with the NIH guide for the Care and Use of Laboratory animals and were approved by the Animal Care and Use Committee of Northwestern University.

### Reagents

The NIH Clinical Collection (NCC; Developmental Therapeutics Program, NCI/NIH, Bethesda, Maryland) was aliquotted robotically from 10 mM stocks at the High Throughput Analysis Laboratory of Northwestern University. Simvastatin, fluvastatin, lovastatin, atorvastatin, pravastatin, H1152, fasudil and hydroxyfasudil were products of EMD Millipore (Billerica, MA). Geranylgeraniol, farnesol, cerivastatin (other than in the NCC collection), laminin, Penicillin/Streptomycin stock (10,000 units Penicillin and 10 mg streptomycin per ml), DMEM/Hams F12 (1:1) medium, L-glutamine, 45% glucose, DNAse I (10 μg/ml), and DMSO were purchased from Sigma-Aldrich (St. Louis, MO). Brain derived neurotrophic factor (BDNF) was purchased from Promega (Madison, WI, USA), Neurotrophic factor 3 (NT3) was purchased from Peprotech (Rocky Hill, NJ, USA) and Bone morphogenic protein 4 (BMP4) was purchased from R&D Systems (Minneapolis, MN). N2 mix, Alexa Fluor 594 conjugated F(ab’)_2_ fragment of goat anti mouse IgG (H + L) and Nuclear Yellow were purchased from Invitrogen/Life Technologies (Carlsbad, CA, USA). Dispase (neutral protease) was purchased from Worthington Biochemical Corporation (Lakewood, New Jersey). Fetal bovine serum (FBS) was purchased from Hyclone (Logan, Utah) and heat inactivated before use. Poly-D-Lysine coated plates were the product of Becton, Dickinson & Co (Franklin Lakes, New Jersey). The monoclonal antibody TuJ1 was purchased from Covance (Princeton, NJ).

### Software

HCA Vision^58^ was purchased from CSIRO Computational Informatics (North Ryde, Australia); Graphpad Prism is a product of Graphpad Software Inc. (La Jolla, CA). Cellomics software is a product of Thermo Scientific. Custom utility software for simplifying data transfer between programs and for montaging images were contributed by Abraham Rozental.

### Cultures

Cultures were prepared by a modification of the procedure previously reported and modified^21^,^31^,^32^,^33^. To increase the number of cultures produced from one dissecting session while maintaining consistency in the preparations, the number of animals was increased to 8 (16 cochleas, a practical limit) and the volume of the cultures was decreased to 50 μl. Cells derived from the equivalent of 0.073 ganglia were plated in each well. These changes allowed the material from each dissection session to be cultured in 192 wells of a standard 384 well plate. Each dissection session could accommodate 3 controls and 45 compounds in quadruplicate. *Briefly*: The cochlear capsule was opened and the tissue was treated with Dispase, 5 units/ml. After washing, the cochlea was removed from its capsule. The cochlear epithelium, spiral ligament and stria were dissected away. Spiral ganglia with attached modiolar tissue, spiral lamina and limbus were dissociated in complete medium (DMEM/Hams F12(1:1), 2 mM L glutamine, N2 mix (1:100), 45% glucose (0.63 ml/100 mls DMEM/Hams F12.) and routinely plated on poly-D-lysine/laminin coated 384 well plates. In early experiments the poly-D-lysine was manually applied before the laminin. In later experiments, commercial plates precoated with poly-D-lysine were manually overlaid with laminin. Cultures were maintained in standard medium containing complete medium, neurotrophins (final concentrations: BDNF and NT3, 10 ng/ml each), 10% heat inactivated FBS, penicillin (100 units/ml) and streptomycin (100 μg/ml). Candidate compounds in the screen were dissolved in DMSO, then diluted in medium and were added to the wells 22 hours after plating (final concentration 8.25 μM, 0.17% DMSO). Pravastatin, H1152, fasudil (100 μM final), and hydroxyfasudil (100 μM final) were dissolved in water before medium dilution; farnesol was dissolved in ethanol for medium dilution. Cultures were fixed 24 hours later with 4% paraformaldehyde in 0.1 M sodium phosphate buffer, pH 7.2 for 45 minutes. Conditions were tested in quadruplicate. Each plate had its own control cultures. Negative controls were the vehicles DMSO (0.17%) and water (0.17%) and when farnesol was tested, ethanol (0.17%). As we reported (22), the Rho kinase inhibitor H1152 increases neurite length in these cultures and we therefore used it at 16.6 μM as a positive control. All compounds (440) were assayed on cultures maintained in the standard composition of media. A subset of the compound library (160 compounds) was re-assayed in a separate medium that additionally contained the compound BMP4 (final concentration, 25 ng/ml), which we demonstrated to cause shorter neurites (31). Experiments comparing Rho kinase inhibitors H1152, fasudil and hydroxyfasudil (Fig. 2d) were carried out in BMP4 medium and fixed after 4 days.

### Immunolabeling

Fixed cultures were immunolabeled for the neural βIII tubulin using the mouse monoclonal antibody TuJ1 and Alexa Fluor 594 tagged secondary antibody as described for immunofluorescent labeling^32^. Nuclei were visualized with Nuclear Yellow. The labeled cultures were preserved with one drop of DAKO fluorescent mounting medium in each well.

### Imaging

The immunolabeled 384 well plates were loaded into a Cellomics ArrayScan VTi for image acquisition. The ArrayScan contains a Zeiss epifluorescence microscope housed inside a robotic platform to automatically capture images from a designated number of fields for each well. Immunolabeled wells were imaged with a 10x objective with two filter sets targeting nuclei (Nuclear yellow) and the immunostained neurons (βIII-tubulin, Alexafluor 594). Sixteen non-overlapping fields in each well were acquired with a high resolution charge-coupled device (CCD) camera, about 80% of the surface area of the well.

### Neurite Length Measurements

Initially, neurites were automatically detected and measured using Cellomics software and the length was expressed in μm. However, this version of the software was inconsistent in detecting very thin neurites in our preparations. We used HCA Vision^58^ for analyzing neurite lengths in the screen, and the resulting neurite lengths are expressed in pixels. The pixel measurement of the longest uninterrupted path of a neurite for each neuron in the well was acquired. Neurite lengths for the population in each well were plotted using GraphPad Prism Software. For each well, the population of neurite lengths was plotted as a cumulative percent histogram, starting at 20 pixels length and using a bin size of 1 pixel. The histograms for the replicate wells were averaged and plotted with SEM. The positive control, H1152 (16.5 μM) was plotted first from 0–100%. The graph was then reduced to the portion representing 25–75 percentile. All other graphs were plotted on the same axes. Negative control graphs (the vehicles DMSO and water) were always higher and to the left of the positive control graphs. Compounds that generated histograms that were similar to the positive control were retested in new experiments. Compounds that continued to generate histograms similar to the positive control were retested at different concentrations. Dose response curves were also carried out on a panel of several statins. In some experiments, the statins were added together with geranylgeraniol (10 μM) or farnesol (10 μM).

To evaluate the average lengths of the longest neurites, the ten longest neurites in quadruplicate wells of water, DMSO, H1152 and cerivastatin treated cultures were analyzed. The GraphPad Prism program was used to identify and remove outliers by the ROUT method in each group. Quadruplicate wells were averaged in each experiment. Then the mean was taken of the averages from 5 experiments.

### Neuron Number

Number of neurons was counted manually on the 16 images from each well.

## Additional Information

**How to cite this article**: Whitlon, D. S. *et al.* Novel High Content Screen Detects Compounds That Promote Neurite Regeneration From Cochlear Spiral Ganglion Neurons. *Sci. Rep.* **5**, 15960; doi: 10.1038/srep15960 (2015).

## Supplementary Material

Supplementary Information
