# Supplementary material for: Novel High Content Screen Detects Compounds That Promote Neurite Regeneration from Cochlear Spiral Ganglion Neurons
Source: Sci Rep. 2015 Nov 2;5:15960. doi: 10.1038/srep15960 (PMC4629150; doi:10.1038/srep15960)
Supplement: Supplementary Information [file srep15960-s1.pdf]

Supplemental Information
Table 1

Novel High Content Screen Detects Compounds That

Promote Neurite Regeneration from Cochlear Spiral Ganglion Neurons

Donna S. Whitlon[1,2,3*], Mary Grover[1], Sara F. Dunne[4], Sonja Richter[1], Chi-Hao Luan[4]

and Claus-Peter Richter[1,2,5]

All compounds assayed at 8.3µM
Compounds in italics were also assayed in BMP4 medium
Nalbuphine
Raclopride
Zacopride
SKF 83566
3'-deoxydenosine
AM 404
PILOCARPINE HYDROCHLORIDE
NIFEDIPINE
Flurbiprofen
3-HYDROXY-1,2-DIMETHYL-4(1H)-PYRIDONE
LOXAPINE SUCCINATE
d-3-Methoxy-N-methylmorphinan hydrobromide
Duloxetine
Glycine, N-[2-[(acetylthio)methyl]-1-oxo-3-phenylpropyl]-,phenylmethyl ester [CAS]
Benzeneacetic acid, 2-[(2,6-dichlorophenyl)amino]-, monosodium salt [CAS]
PROGESTERONE
FAMOTIDINE
SR 57,227A
Pancuronium
METRONIDAZOLE
Benzeneacetic acid, Alpha-(hydroxymethyl)-, 9-methyl-3-oxa-9-azatricyclo[3.3.1.02,4]non-7-yl ester, [7(S)-(1Alpha,2ÃƒÂ¿,4ÃƒÂ¿,5Alpha,7ÃƒÂ¿)]- [CAS]
Benzeneacetonitrile, Alpha-[3-[[2-(3,4-dimethoxyphenyl)ethyl]methylamino]propyl]-3,4-dimethoxy-Alpha-(1-methylethyl)-, (R)- [CAS]
Benzeneethanamine, N,Alpha-dimethyl-N-2-propynyl-(R)- [CAS]
Capsaicin
SALBUTAMOL SULFATE
(Â±)-Vesamicol hydrochloride
Picrotin - Picrotoxinin
Terazosin
diphenylcyclopropenone
4-Thiazolidinecarboxylic acid, 2-oxo-, (R)- [CAS]
Mesoridazine
3(2H)-Pyridazinone, 6-[4-(difluoromethoxy)-3-methoxyphenyl]- [CAS]
10H-Phenothiazine, 2-chloro-10-[3-(4-methyl-1-piperazinyl)propyl- [CAS]
1H-Cyclopenta[b]quinolin-9-amine, 2,3,5,6,7,8-hexahydro-, monohydrochloride- [CAS]
CLOTRIMAZOLE
LORATADINE
PHENELZINE SULFATE SALT
Riluzole
Naltrindole
Nornicotine
Bifemelane
CGS 15943
Cinanserin
Cisapride
Indatraline
TRAZODONE HYDROCHLORIDE
Prazosin
URAPIDIL HYDROCHLORIDE
(-)-Cotinine
D-CYCLOSERINE
Fluvoxamine
Doxepin
TRIFLUOPERAZINE DIHYDROCHLORIDE
(+)-3-HYDROXY-N-METHYLMORPHINAN D-TARTRATE
L-Ornithine, N5-[imino(methylamino)methyl]-[CAS]
Maprotilline HCl
Pizotyline
BETA-ESTRADIOL
N,N'-DIACETYL-1,6-DIAMINOHEXANE
DIPHENHYDRAMINE HYDROCHLORIDE
Galanthamine
Ifenprodil
TETRAETHYLTHIURAM DISULFIDE
Piribedil
KETOCONAZOLE
TRIPELENNAMINE HYDROCHLORIDE
Pyrazinecarboxamide, 3,5-diamino-N-(aminoiminomethyl)-6-chloro- [CAS]
9-AMINO-1,2,3,4-TETRAHYDROACRIDINE HYDROCHLORIDE
ETHYNYLESTRADIOL
2(1H)-Pyrimidinone, 4-amino-1-ÃƒÂ¿-D-arabinofuranosyl- [CAS]
L-Glutamic acid, N-[4-[[(2,4-diamino-6-pteridinyl)methyl]methylamino]benzoyl]- [CAS]
TFMPP
Pramipexole
LIDOCAINE
Indomethacin
LY 171883
Paroxetine

Epigallocatechin gallate
5-Amino-2-hydroxy-benzoic acid
Oxiranecarboxylic acid, 2-[6-(4-chlorophenoxy)hexyl]-, ethyl ester- [CAS]
Cephalexin monohydrate
PIDOTIMOD
RAMIPRIL
LEVOFLOXACIN
19-Nortestosterone
NIZATIDINE
5-FLUOROCYTOSINE
Trileptal
TROXIPIDE
ACTARIT
AZELASTINE HCl
TOCAINIDE
TAXIFOLIN-(+/-)
FENPIVERINIUM BROMIDE
CEFATRIZINE PROPYLENE GLYCOL
IDEBENONE
LEVOSULPIRIDE
Pemoline
LETROZOLE
MEROPENEM
ORLISTAT
ONDANSETRON HCl
LEVONORGESTREL
CETRAXATE HCl
Alprazolam
LAMOTRIGINE
CROTAMITON
AMFEBUTAMONE HCl
ALFUZOSIN
Amisulpride
LOFEPRAMINE
PEROSPIRONE HCl
DOCETAXEL
HONOKIOL
TOLTERODINE TARTRATE
CARMOFUR
PAROXETINE
OLMESARTAN MEDOXOMIL
LOSARTAN Potassium
TEMOZOLOMIDE
Methyltestosterone
TOSUFLOXACIN TOSYLATE
MECILLINAM
ATOMOXETINE HCl
ARTESUNATE
ITRACONAZOLE
CEFPODOXIME PROXETIL
BUFLOMEDIL HYDROCHLORIDE
4-Chloro-N-(2-morpholin-4-yl-ethyl)-benzamide
HALOMETASONE MONOHYDRATE
TRICLABENDAZOLE
ROFECOXIB
BISOPROLOL FUMARATE
EZETIMIBE
TIAGABINE HCl
IDARUBICIN HCl
FLUBENDAZOLE
TACROLIMUS
VALACICLOVIR HYDROCHLORIDE
CLARITHROMYCIN
ARIPIPRAZOLE
TRIMEBUTINE MALEATE
Mestanolone
NISOLDIPINE
PICEID
1-(2-Methyl-5-nitro-imidazol-1-yl)-propan-2-ol
NIFEKALANT HCl
NATEGLINIDE
MEGESTROL ACETATE
ORMETOPRIM
ZILEUTON
STAVUDINE
GABEXATE MESYLATE
OXICONAZOLE NITRATE
KITASAMYCIN
FAMCICLOVIR
(Â±)-Sotalol hydrochloride
RUFLOXACIN HCl
TAXIFOLIN-(+)

ALOSETRON HCl
BUPROPION HYDROCHLORIDE
IRSOGLADINE MALEATE
ACARBOSE
BENPROPERINE PHOSPHATE
PHENPROBAMATE
MEMANTINE HYDROCHLORIDE
Carvedilol
LOMIFYLLINE
PAZUFLOXACIN
MIGLITOL
TRANILAST
OLANZAPINE
Nefazodone
MOXIFLOXACIN HCl
NELFINAVIR MESYLATE
PRAVASTATIN Sodium
TOPOTECAN HCL
LEVETIRACETAM
PRAMIPEXOLE HCl
RISPERIDONE
PIOGLITAZONE HCl
CILASTATIN Na
ARGATROBAN
VALDECOXIB
NAFTOPIDIL
Nobiletin
FINASTERIDE
ZOLPIDEM TARTRATE
Viramune
TOPIRAMATE
VORICONAZOLE
FENOLDOPAM MESYLATE
ROSIGLITAZONE MALEATE
ESCITALOPRAM OXALATE
ZERANOL
LATANOPROST
2',3'-DIDEOXYINOSINE
Sertraline
CALCIPOTRIOL
EPIRUBICIN HYDROCHLORIDE
BICALUTAMIDE
BENIDIPINE HCl
AMLEXANOX
CERIVASTATIN Na
ICARIIN
METHYLANDROSTENEDIOL
TRIPTOLIDE
ROSIGLITAZONE HCl
FTORAFUR
OLIGOMYCIN C
BENAZEPRIL HCl
Oxymetholone
IPRIFLAVONE
OXAPROZIN
ROLIPRAM
MOSAPRIDE CITRATE
Isoquercitrin
FLUMAZENIL
OZAGREL HCl
HYPEROSIDE
RIFABUTIN
ESMOLOL HYDROCHLORIDE
TADALAFIL
Modafinil
DOXORUBICIN HYDROCHLORIDE
MOXONIDINE HCl
Nitrazepam
PEFLOXACIN MESYLATE
VENLAFAXINE HCl
PANTOPRAZOLE SODIUM SALT
FLUTICASONE PROPIONATE
INDINAVIR SULPHATE
MIDAZOLAM HCl
LAMIVUDINE
PROCARBAZINE HCl
ESOMEPRAZOLE Mg
SULFASALAZINE
TORASEMIDE
TROPISETRON HCl
Ranolazine dihydrochloride
NITRENDIPINE

SAQUINAVIR MESYLATE
BIFONAZOLE
SUMATRIPTAN SUCCINATE
EXEMESTANE
NITAZOXANIDE
Diazepam
QUETIAPINE HEMIFUMARATE
RUTIN
PENCICLOVIR
CALCITRIOL
DIPHENOXYLATE
Felbamate
DROPERIDOL
Pentoxifylline
TORADOL
RITONAVIR
VINORELBINE BITATRATE
LINEZOLID
LOMERIZINE DiHCl
EFAVIRENZ
IRBESARTAN
REPAGLINIDE
Ethylestrenol
PTEROSTILBENE
ROXATIDINE ACETATE HCl
DEXBROMPHENIRAMINE MALEATE
ANAGRELIDE HCl
TEGASEROD MALEATE
MILRINONE
LEVOCETIRIZINE
Citalopram hydrobromide
TICLOPIDINE HCl
LOXOPROFEN SODIUM
ZAFIRLUKAST
TERBINAFINE HCl
ISRADIPINE
VALSARTAN
Piroxicam
GLYCOPYRROLATE
Physostigmine
LOBELINE HYDROCHLORIDE
DOXYLAMINE SUCCINATE
Milnacipran
5-fluoro-2-pyrimidone
Chlorpheniramine
DOFETILIDE
FORMOTEROL FUMARATE DIHYDRATE
RIZATRIPTAN BENZOATE
RIFAPENTINE
LOTEPREDNOL ETABONATE
ENALAPRILAT
Donepezil
Nimetazepam
NICORANDIL
TELMISARTAN
ITOPRIDE HCl
RIFAXIMIN
MONTELUKAST Na
2',3'-DIDEOXYCYTIDINE
1H-Imidazol-2-amine, N-(2,6-dichlorophenyl)-4,5-dihydro- [CAS]
6H-Pyrido[2,3-b][1,4]benzodiazepin-6-one, 11-[[2-[(diethylamino)methyl]-1-piperidinyl]acetyl]-5,11-dihydro- [CAS]
1H-Indole-2-propanoic acid, 1-[(4-chlorophenyl)methyl]-3-[(1,1-dimethylethyl)thio]-Alpha,Alpha-dimethyl-5-(1-methylethyl)- [CAS]
1H-Imidazole-5-carboxylic acid, 1-(1-phenylethyl)-, ethyl ester, (R)- [CAS]
Acetamide, 2-amino-N-(1-methyl-1,2-diphenylethyl)-, (+/-)- [CAS]
Altanserin
BETAXOLOL HYDROCHLORIDE
IDARUBICIN
Azasetron
GR 89696
DELTA1-HYDROCORTISONE 21-HEMISUCCINATE SODIUM SALT
DIAZOXIDE
2-CHLOROADENOSINE
ORNIDAZOLE
1,1-DIMETHYL-4-PHENYLPIPERAZINIUM IODIDE
PIRENPERONE
MESTRANOL
2-(2-AMINOETHYL)PYRIDINE
BENACTYZINE HYDROCHLORIDE
DICHLOROACETIC ACID
BESTATIN
TOREMIFENE CITRATE
GOSERELIN ACETATE

SECOISOLARICIRESINOL
RALTITREXED
DOXAPRAM HYDROCHLORIDE
RU 24969
Brucine
TRYPTOLINE
FLUPHENAZINE HYDROCHLORIDE
PALONOSETRON HCl
NAPROXEN SODIUM
MEPIVACAINE HYDROCHLORIDE
3-[3,5-DIBROMO-4-HYDROXYBENZOYL]-2-ETHYLBENZOFURAN
NIMODIPINE
ROLITETRACYCLINE
MEPIRIZOLE
6-AZAURIDINE
ReichsteinÂ's substanceÂ S
3-PYRIDINEMETHANOL
Haloperidol
Stiripentol
Fluperlapine
OXYPHENONIUM BROMIDE
Homoveratrylamine
TINIDAZOLE
XANTHINOL NICOTINATE
SYNEPHRINE
RESVERATROL
MALTOL
6-AMINOINDAZOLE
ENROFLOXACIN
DEHYDROCHOLIC ACID
CEFACLOR
1-BENZYLIMIDAZOLE
DULOXETINE HCl
VARDENAFIL CITRATE
ROPIVACAINE HCl
ANASTROZOLE
KETOTIFEN FUMARATE
MEDROXYPROGESTERONE
Pinacidil monohydrate
7-NITROINDAZOLE
5-Methoxytryptamine
GRANISETRON HCl
Rimcazole
Nafadotride
DESOXIMETASONE
DEXCHLORPHENIRAMINE MALEATE
Guanidine, N-cyano-N'-(1,1-dimethylpropyl)-N''-3-pyridinyl- [CAS]
L-694,247
AM-251
HTMT
Benzo[a]phenanthridine-10,11-diol, 5,6,6a,7,8,12b-hexahydro-, trans- [CAS]
Methanesulfonamide, N-[4-[[1-[2-(6-methyl-2-pyridinyl)ethyl]-4-piperidinyl]carbonyl]phenyl]-, dihydrochloride [CAS]
2H-Indol-2-one, 1,3-dihydro-1-phenyl-3,3-bis(4-pyridinylmethyl)- [CAS]
Beclomethasone
OMEPRAZOLE
DOLASETRON MESYLATE
Zolmitriptan
TREMULACIN
DACTINOMYCIN
Tramadol
CHLORDIAZEPOXIDE
CEFIXIME TRIHYDRATE
CEFDINIR
LOFEXIDINE HCl
BALSALAZIDE
OLOPATADINE HCl
ITAVASTATIN Ca
CORTISONE
CYPROHEPTADINE HYDROCHLORIDE
HOMOHARRINGTONINE
CORTICOSTERONE
VECURONIUM BROMIDE
TIBOLONE
NICOTINAMIDE
NIALAMIDE
VINDESINE SULFATE
VINCRISTINE SULFATE
LACIDIPINE
MIRTAZAPINE
AMPIROXICAM
GLIMEPIRIDE
AMLODIPINE BASE

RABEPRAZOLE
CLOFAZIMINE
IRINOTECAN HCl)trihydrate)
LANSOPRAZOLE
8-Chloro-11-piperidin-4-ylidene-6,11-dihydro-5H-benzo[5,6]cyclohepta[1,2-b]pyridine
1,3,5(10)-ESTRATRIEN-3-OL-17-ONE SULPHATE, SODIUM SALT
MIFEPRISTONE
Etoposide
Sibutramine
Clobenpropit
HUPERZINE A
SIBUTRAMINE HCl
Lorazepam
8-Azaspiro[4.5]decane-7,9-dione, 8-[2-[[(2,3-dihydro-1,4-benzodioxin-2-yl)methyl]amino]ethyl]-, monomethanesulfonate [CAS]
Adenosine, N-(2-hydroxycyclopentyl)-, (1S-trans)- [CAS]
AMIODARONE HYDROCHLORIDE
mevastatin
IMATINIB MESYLATE
Metylperon
PARECOXIB Na
PERGOLIDE MESYLATE SALT
ATRACURIUM BESYLATE
ARTEMETHER
EBSELEN
CGS 12066B
TELITHROMYCIN
CCPA
PD 81723
Stanozolol
Zaleplon
Prostaglandin E1
Testosterone
DEHYDROEPIANDROSTERONE
SDM25N
Thiophene, 5-bromo-2-(4-fluorophenyl)-3-[4-(methylsulfonyl)phenyl]- [CAS]
5-Nonyloxytryptamine
Salmeterol
SB 205607
R(+)-SCH-23390 hydrochloride
